# Primary Tumor Site Affects Survival in Patients with Gastroenteropancreatic and Neuroendocrine Liver Metastases

**DOI:** 10.1155/2019/9871319

**Published:** 2019-03-12

**Authors:** John F. Tierney, Jennifer Poirier, Sitaram Chivukula, Sam G. Pappas, Martin Hertl, Erik Schadde, Xavier Keutgen

**Affiliations:** ^1^Division of Surgical Oncology, Department of Surgery, Rush University Medical Center, Chicago, IL, USA; ^2^Division of Transplant, Department of Surgery, Rush University Medical Center, Chicago, IL, USA; ^3^Cantonal Hospital Winterthur, Department of Surgery, Winterthur, Zurich, Switzerland; ^4^University of Zurich, Institute of Physiology, Zurich, Switzerland; ^5^Division of General Surgery, Department of Surgery, The University of Chicago Medical Center, Chicago, IL, USA

## Abstract

**Background:**

Gastroenteropancreatic neuroendocrine tumors (GEP-NETs) are commonly present with metastatic disease, and the liver is the most frequent metastatic site. Herein, we studied whether primary tumor site affects survival in patients with GEP-NETs and liver metastases (NELM). As a secondary endpoint, we studied whether extrahepatic disease and surgical resection impact survival in this patient population.

**Methods:**

Patients with NELM diagnosed from 2006 to 2014 were identified from the National Cancer Database. Kaplan-Meier curves and nested Cox proportional hazards were used to assess variables associated with survival.

**Results:**

2947 patients with well- or moderately differentiated GEP-NETs and NELM met the inclusion criteria for this study. Patients with small bowel NETs survived the longest of all GEP-NETs with NELM (median not reached). Rectal and gastric NETs with NELM had the shortest survival (median 31 months). Patients with extrahepatic metastases who underwent any operation survived longer than those managed nonoperatively (median survival 38.7 months vs. 18.6 months, *p* = 0.01). On multivariable analysis, operations on the primary tumor and distant metastatic site (HR 0.23-0.43 vs. no surgery), treatment at an academic/research hospital, Charlson comorbidity index of 0, no extrahepatic metastases, and younger age were associated with prolonged survival (*p* < 0.01).

**Conclusions:**

Primary tumor site affects survival in patients with GEP-NETs and NELM. Surgical resection seems beneficial for all GEP-NETs with NELM, even in the presence of extrahepatic metastases.

## 1. Introduction

Gastroenteropancreatic neuroendocrine tumors (GEP-NETs) were initially thought to be small indolent tumors, when first described 100 years ago, but are now recognized to have malignant potential, with 40-50% of patients developing distant metastases [1]. The presence of metastatic disease is strongly associated with survival for GEP-NETs [2]. The liver is the most common metastatic site for these tumors, and 80% of patients eventually succumb to liver failure due to metastatic tumors [3].

Neuroendocrine tumors (NETs) are one of the few tumor types in which debulking surgery—most commonly performed to remove tumor from the liver—is a recommended treatment for metastatic disease [4, 5]. Current European Neuroendocrine Tumor Society (ENETS) guidelines recommend resection of liver metastases from well- or moderately differentiated tumors if surgery removes at least 90% of the tumor burden, and some centers in the Unites States have further lowered the threshold for debulking surgery to 70% with similar outcomes [1, 6].

Although the association of liver metastases with mortality in patients with GEP-NETs is well-established, it is unknown whether primary tumor site is associated with prognosis among patients with neuroendocrine liver metastases (NELM). Additionally, it remains controversial if patients with both hepatic and extrahepatic metastases benefit from operative management, as is the case in the carefully selected patient with colorectal liver metastases [7]. We therefore used the National Cancer Database (NCDB) to examine survival in patients with grade 1 and 2 GEP-NETs and NELM to determine whether primary tumor site influences survival and also to examine the survival of GEP-NET patients with extrahepatic metastases with and without surgical resection.

## 2. Methods

The NCDB is a joint project of the Commission on Cancer of the American College of Surgeons and the American Cancer Society. It represents the largest database of cancer patients in the United States, comprising about 70% of all patients newly diagnosed with cancer, and contains information regarding cancer treatments within the first six months of diagnosis [8, 9]. The NCDB and the hospitals participating in the NCDB are the source of the de-identified data used herein; they have not verified and are not responsible for the statistical validity of the data analysis or the conclusions derived by the authors. This study was exempt from IRB review because the NCDB is a public database that does not contain personally identifiable patient information.

Patients diagnosed with neuroendocrine tumors in the colon, pancreas, rectum, small intestine, and stomach between 2004 and 2014 were identified from the NCDB according to tumor location and histology code. Patients with liver metastases at the time of diagnosis were identified by the “CS_METS_ DX_LIVER” variable. Only patients with grade 1 and 2 tumors were included. Grade for gastrointestinal NETs was determined by histology code. The codes 8240 and 8249 (well- or moderately differentiated tumors) were defined as grade 1 or 2 tumors and 8013, 8041, and 8246 (poorly differentiated tumors), as grade 3 tumors, and therefore excluded from this study. Grade for pancreatic NETs was determined by the “GRADE” variable and was classified as grade 1 or 2, grade 3, and missing. Pancreatic NET patients with grade 3 tumor or missing information on grade were excluded.

For the primary objective of determining whether primary tumor site influences survival among patients with NELM, Kaplan-Meier survival curves were created for patients with localized disease and with NELM for each site (colon, pancreas, rectum, small intestine, and stomach) and compared for each (localized and NELM) across sites using the Mantel-Haenszel tests.

The following variables were then examined individually to determine whether they were associated with survival among all patients with NELM: hospital type, age, sex, race, ethnicity, Charlson comorbidity index (CCI), radiation therapy status, chemotherapy status, presence of extrahepatic metastases, and surgical approach. Surgical approaches included primary tumor surgery, distant metastatic site surgery, primary tumor and distant site surgery, and no surgery. Debulking operations were defined as operations on both primary and metastatic sites. Differences in survival curves were compared with the Mantel-Haenszel tests, and *p* values were adjusted using the Benjamini-Hochberg procedure.

Nested Cox proportional hazard models were created using variables significant on univariable analysis, compared and tested for proportionality. Initial models failed the proportionality assumption, indicating that at least one variable interacted with time; the data were therefore split at 22 months to incorporate this interaction with time, and the subsequent models passed the proportionality assumption. Nested models including the time interaction were subsequently compared using the likelihood ratio tests.

Finally, Kaplan-Meier curves were created comparing patients with and without extrahepatic metastases and surgical to nonsurgical patients. These curves were compared using the Mantel-Haenszel tests.

*p* values less than or equal to 0.05 were considered significant. All analyses were conducted in R, 3.3.2 [10].

## 3. Results

2947 patients with NELM were identified from the NCDB and included for analysis in this study.

**Figure 1:**
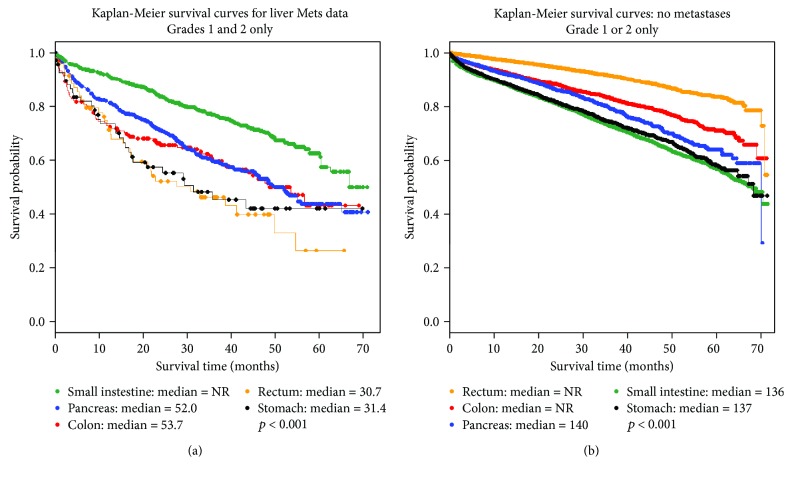
Survival curve by primary tumor site for patients with NELM (a) and stage I-III disease (b).

Survival in patients with NELM differed according to primary tumor site. Median survival for patients with small intestinal primary tumors was not reached; these patients survived significantly longer than patients with any other primary tumor site (*p* < 0.001). Patients with pancreatic NETs survived a median of 52.0 months, significantly longer than those with rectal NETs, who survived a median of 30.7 months (*p* = 0.01) (Table 1) (Figure 1(a)). Patients with colonic NETs survived 53.7 months and those with stomach NETs survived 31.4 months, but there were no significant differences in survival between colonic, rectal, and stomach primary tumor sites or between pancreatic NETs and colonic and stomach NETs (*p* = 0.09-0.70).

In comparison, patients with localized NETs (stage I-III, 47,303 patients identified) had median survivals ≥136 months, regardless of primary tumor site (Figure 1(b)). Patients with rectal NETs survived significantly longer (median not reached) than those with tumors in any other site (*p* < 0.001), and patients with colonic NETs survived longer (median not reached) than those with pancreatic, small intestinal, or gastric NETs (*p* < 0.001).

**Figure 2:**
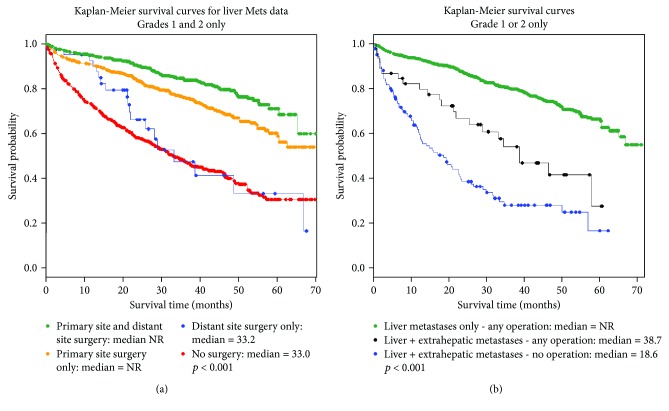
Kaplan-Meier curves by surgical treatment for patients with NELM (a) and by metastatic site and operative vs. nonoperative management (b).

Among all patients with NELM, 644 (21.8%) underwent an operation on both the primary tumor site and a distant metastatic site (debulking), 625 (21.2%) underwent primary tumor resection only, 41 (1.4%) underwent resection of metastases only, and 878 (29.8%) did not have an operation. Debulking operations were associated with prolonged survival on univariable analysis compared to all other groups (median survival not reached; *p* < 0.001) (Figure 2(a)). Additional factors associated with prolonged survival on univariable analysis included treatment at an academic/research hospital, younger age, lower CCI, absence of chemotherapy, and lack of extrahepatic metastases (Table 2). Debulking operations remained significantly associated with prolonged survival on multivariable analysis (HR 0.23-0.43 when compared to no surgery, *p* < 0.001). Other significant factors associated with prolonged survival identified on multivariable analysis included treatment at an academic/research hospital when compared to all other hospital types, CCI of 0 when compared to all other CCI scores, absence of extrahepatic metastases, and younger patient age (Table 3).

There were significant differences in survival according to primary tumor site among patients who had operations on both the primary tumor site and a distant metastatic site. Patients with small intestinal NETs who had debulking operations survived significantly longer (median not reached) than patients with either pancreatic NETs (65.1 months) or colonic NETs (44.8 months) (*p* < 0.001). Patients with pancreatic NETs who underwent debulking operations survived significantly longer than those with colonic NETs (*p* < 0.001). An insufficient number of patients with gastric or rectal NETs underwent debulking surgery for these patients to be included in the analysis.

Subgroup analysis was performed to evaluate the effect of an operation on survival in NELM patients with extrahepatic metastases. Patients with extrahepatic metastases who underwent any operation survived a median of 38.7 months, shorter than patients with liver metastases alone who underwent an operation (median not reached) but significantly longer than patients with extrahepatic disease who did not have an operation (18.6 months, *p* < 0.001) (Figure 2(b)).

## 4. Discussion

In this study, we demonstrated that among GEP-NETs patients with NELM, significant differences in survival exist according to primary tumor location.

Not surprisingly, patients with localized (stage I-III) GEP-NETs had excellent long-term survival, with median survival times greater than 136 months, consistent with the data from a recent study using the SEER database [11]. Similar to that study, we demonstrated that patients with localized rectal NETs have the longest overall survival (median not reached). The SEER study, which had longer follow-up time than available through the NCDB and included resected patients with any grade tumor, as opposed to all patients with grade 1 or 2 tumors, found that patients with localized pancreatic NETs had the lowest 20-year survival, which was not replicated in the data presented here. This difference could be related to either the shorter follow-up time in the NCDB dataset, our use of overall survival as opposed to the SEER study's use of NET-specific survival, or more likely to the fact that we excluded high-grade NETs [11].

Interestingly, we found that among patients with liver metastases, different primary tumor sites are associated with improved survival. This is the first study to our knowledge that demonstrated that among patients with NELM, those with small intestinal primary tumors have the longest and gastric and rectal NETs have the shortest overall survival. We cannot explain this finding using the data available in the NCDB but suggest that different biological behavior and response to therapies contribute to this observation [12]. Previous studies have demonstrated that small intestinal and pancreatic NETs can be identified by their different genomic profiles, and it is possible that these molecular differences also might affect prognosis [13, 14]. Moreover, response rates of commonly used therapies such as long-acting octreotide analogues also vary according to primary tumor site as demonstrated in previous studies, with pancreatic NETs responding less well to long-acting octreotide then small bowel NETs for example [12, 15]. The difference in prognosis according to primary tumor site seen here also highlights the importance of identifying the location of unknown primary tumors, especially as newer therapies become available [16].

Our secondary objective was to investigate the factors associated with prolonged survival among patients with NELM. In this study, among other factors, debulking operations were associated with prolonged survival, reaffirming the role for an aggressive surgical approach in carefully selected patients with NELM as advocated by many centers [6, 17, 18]. Debulking surgery has been an accepted treatment strategy for stage IV NETs since the 1990s, and the rationale for debulking has evolved from symptom control to prolonging survival over time, but this is the first study using a large national sample to demonstrate a survival benefit for GEP-NET patients with NELM patients who undergo debulking [17, 19]. The precise amount of tumor that should be removed to confer a benefit is controversial, with recommendations ranging from 70% at certain high-volume centers to 90% in the ENETS guidelines [3, 18]. We are unable to comment on a debulking threshold, as the extent of disease or of resection is not reported in the NCDB; prospective studies should be performed to further examine survival after debulking.

The finding that treatment at an academic or research center was independently associated with prolonged survival among all patients with grade 1 or 2 NELM suggests that management of these patients should perhaps be concentrated at high-volume centers, as has been proposed by other authors and in other diseases [20, 21].

Finally, we sought to examine the role of surgical management of patients with extrahepatic metastases. The effect of extrahepatic metastases on survival promises to become a more critical issue for clinicians in determining prognosis and treatment for NELM patients as imaging technologies have improved and ^68^Gallium DOTATATE PET CT is now used more commonly for staging of NETs. In our center's initial experience with ^68^Gallium DOTATATE PET CT, new bone metastases were detected in 18% of patients when compared to conventional imaging such as CT and MRI, thereby vastly expanding the number of patients with known extrahepatic disease [22]. It remains unclear, however, whether the presence of extrahepatic metastases should be a contraindication to operative management. Herein, we found that the presence of extrahepatic metastases was associated with reduced survival among all patients with GEP-NETs and NELM, but patients with extrahepatic metastases who underwent surgery survived significantly longer than those who did not.

We acknowledge that a selection bias could have affected these findings, as patients who underwent surgery might have had less extensive disease either within or outside the liver. The prolonged survival demonstrated among patients who had an operation, however, may indicate that operative management of these patients conveys some benefit in a subset of patients. Two previous studies of patients who underwent liver-directed therapy (resection, debulking, and/or ablation) for neuroendocrine metastases at high-volume centers found that although patients with extrahepatic metastases had worse prognoses than patients with liver metastases alone, they still enjoyed a median survival of up to 87 months [23, 24].

We expect that as imaging technology continues to improve, clinicians will face the challenge of treating patients with extrahepatic metastases more frequently. Further prospective studies should be performed to evaluate the role of operative management in these patients.

There are several limitations to this study, in addition to the inherent data entry errors common to all large database studies. First, this study is retrospective and observational, and therefore, is subject to selection bias. The effects of selection bias are particularly important in the interpretation of the findings that debulking is associated with prolonged survival among all NELM patients and that surgery is associated with prolonged survival among patients with both hepatic and extrahepatic metastases, as discussed above. It is possible that a variable that is unable to be assessed in the NCDB, such as tumor burden or extent of tumor removal during surgery, is responsible for the differences in survival reported here. Operations on the primary and metastatic site, which were classified as debulking operations, could include anything from a liver biopsy to a radical resection of all metastatic foci. The survival benefits described here, however, confirm the experience of high-volume centers described in larger retrospective cohorts, which suggests that optimal management of NELM should, in well-selected patients, include debulking and consideration of surgery in patients with extrahepatic metastases. Second, the NCDB only includes treatment information within the first six months of diagnosis, and does not provide information regarding the type (e.g., long-acting octreotide analogues) or use of specific preoperative, nonoperative, or adjuvant management, which improve outcome [25, 26]. Third, a large amount of patients listed in the NCDB with distant metastases at the time of diagnosis did not have a specific metastatic site listed, and it is likely that some of these patients had liver metastases but were excluded from the study.

Despite these limitations, we have shown that among GEP-NET patients with NELM, those with small intestinal primary tumors have the best overall prognosis that debulking is associated with prolonged survival in these patients and that a subset of patients with extrahepatic metastases might also potentially benefit from operative management.

## Figures and Tables

**Table 1:** Survival by primary tumor site among patients with NELM.

Location of primary site tumor	*N*	Median	95% CI
Small intestine	1009	NR	62.6 – NR
Pancreas	833	52.0	46.0 – 56.0
Colon	206	53.7	38.8 – NR
Rectum	70	30.7	17.0 – 49.9
Stomach	70	31.4	16.8 – NR

**Table 2:** Factors associated with prolonged survival among patients with neuroendocrine liver metastases on univariate analysis.

Variable	*N*	Median survival (months)	95% CI	*p* value
Hospital type				**<0.001**
Community Cancer Program	163	48.5	37.4 – NR	
Comprehensive Community Cancer Program	720	57.8	48.8 – 66.9	
Academic/Research Program	986	NR	60.4 – NR	
Integrated Network Cancer Program	212	48.6	40.3 – 54.7	
Age				**<0.001**
Age < median (61 years)	1157	NR	65.1 – NR	
Age ≥ median (61 years)	1031	44.4	38.6 – 52.0	
Gender				0.99
Female	1034	62.6	54.8 – NR	
Male	1154	60.4	54.9 – NR	
Race				0.23
Asian or Pacific Islander	34	NR	NR – NR	
Black	313	65.1	62.6 – NR	
Other or unknown	48	NR	35.6 – NR	
White	1793	60.2	54.9 – NR	
Hispanic				0.99
Hispanic	88	NR	52.1 – NR	
Non-Spanish; non-Hispanic	2013	60.4	56.0 – NR	
Unknown	87	NR	38.2 – NR	
Charlson comorbidity index				**<0.001**
Charlson 0 or none	1666	60.4	56.7 – NR	
Charlson 1	388	NR	44.1 – NR	
Charlson 2 or more	134	41.9	24.4 – NR	
Radiation therapy				0.36
No radiation therapy	2016	62.6	56.7 – NR	
Radiation therapy	142	49.8	37.1 – NR	
Chemotherapy				**<0.001**
Chemotherapy	543	46.5	35.8 – 54.9	
No chemotherapy	1525	66.9	60.2 – NR	
Extrahepatic metastatic sites				**<0.001**
No extrahepatic metastatic sites	2026	65.1	60.4 – NR	
Extrahepatic metastatic sites	162	22.4	17.0 – 29.8	
Surgery				**<0.001**
No surgery	878	33.0	29.1 – 37.4	
Distant site surgery only	41	33.2	21.9 – 66.9	
Primary site surgery only	625	NR	60.4 – NR	
Primary and distant site surgery	644	NR	65.1 – NR	

**Table 3:** Factors associated with prolonged survival among patients with neuroendocrine liver metastases on multivariable analysis.

Variable	HR	95% CI	*p* value
Hospital type: Community Cancer Program	1.43	1.08 - 1.88	**0.01**
Hospital type: Comprehensive Community Cancer Program	1.24	1.05 - 1.48	**0.01**
Hospital type: Integrated Network Cancer Program	1.46	1.14 - 1.88	**0.003**
Hospital type: Academic/Research Program	1		
Charlson: score 2	1.70	1.24 - 2.32	**<0.001**
Charlson: score 1	1.22	0.96 - 1.54	0.11
Charlson: score 0	1		
Extrahepatic metastases	1.76	1.40 - 2.22	**<0.001**
No extrahepatic metastases	1		

*Before 22 months*			
Age ≥ 61 years	2.18	1.76 - 2.69	**<0.001**
Age < 61 years	1		
Surgery: none	1		
Surgery: distant site only	0.57	0.30 - 1.08	0.09
Surgery: primary site only	0.36	0.28 - 0.46	**<0.001**
Surgery: debulking	0.23	0.17 - 0.31	**<0.001**

*After 22 months*			
Age ≥ 61 years	1.37	1.05 - 1.78	**0.02**
Age < 61 years	1		
Surgery: none	1		
Surgery: distant site only	1.05	0.46 - 2.43	0.90
Surgery: primary site only	0.58	0.42 - 0.78	**<0.001**
Surgery: debulking	0.43	0.31 - 0.61	**<0.001**

## Data Availability

The data used for this study was obtained from the National Cancer Database. It may be requested from the National Cancer Database during the biannual Participant User Files application period.

## Additional Points

*Synopsis*. Patients with small intestinal primary NETs have the best prognosis of all patients with neuroendocrine liver metastases. Debulking operations are associated with prolonged survival for all GEP NET patients.
